# Salivary Gland Proteome Analysis Reveals Modulation of Anopheline Unique Proteins in Insensitive Acetylcholinesterase Resistant *Anopheles gambiae* Mosquitoes

**DOI:** 10.1371/journal.pone.0103816

**Published:** 2014-08-07

**Authors:** Sylvie Cornelie, Marie Rossignol, Martial Seveno, Edith Demettre, François Mouchet, Innocent Djègbè, Philippe Marin, Fabrice Chandre, Vincent Corbel, Franck Remoué, Françoise Mathieu-Daudé

**Affiliations:** 1 Maladies Infectieuses et Vecteurs, Ecologie, Génétique, Evolution et Contrôle (MIVEGEC), UMR IRD 224-CNRS 5290-UM1-UM2, Institut de Recherche pour le Développement (IRD), Montpellier, France; 2 Institut de Génomique Fonctionnelle (IGF), UMR CNRS 5203-INSERM 661-UM1-UM2, Montpellier, France; 3 Maladies Infectieuses et Vecteurs, Ecologie, Génétique, Evolution et Contrôle (MIVEGEC), UMR IRD 224-CNRS 5290-UM1-UM2, Institut de Recherche pour le Développement (IRD), Centre de Recherche Entomologique de Cotonou (CREC), Cotonou, Bénin; Kansas State University, United States of America

## Abstract

Insensitive acetylcholinesterase resistance due to a mutation in the acetylcholinesterase (ace) encoding *ace-1* gene confers cross-resistance to organophosphate and carbamate insecticides in *Anopheles gambiae* populations from Central and West Africa. This mutation is associated with a strong genetic cost revealed through alterations of some life history traits but little is known about the physiological and behavioural changes in insects bearing the *ace-1^R^* allele. Comparative analysis of the salivary gland contents between *An. gambiae* susceptible and *ace-1^R^* resistant strains was carried out to charaterize factors that could be involved in modifications of blood meal process, trophic behaviour or pathogen interaction in the insecticide-resistant mosquitoes. Differential analysis of the salivary gland protein profiles revealed differences in abundance for several proteins, two of them showing major differences between the two strains. These two proteins identified as saglin and TRIO are salivary gland-1 related proteins, a family unique to anopheline mosquitoes, one of them playing a crucial role in salivary gland invasion by *Plasmodium falciparum* sporozoites. Differential expression of two other proteins previously identified in the *Anopheles* sialome was also observed. The differentially regulated proteins are involved in pathogen invasion, blood feeding process, and protection against oxidation, relevant steps in the outcome of malaria infection. Further functional studies and insect behaviour experiments would confirm the impact of the modification of the sialome composition on blood feeding and pathogen transmission abilities of the resistant mosquitoes. The data supports the hypothesis of alterations linked to insecticide resistance in the biology of the primary vector of human malaria in Africa.

## Introduction

Malaria remains one of the most important public health problems worldwide. Due to its high anthropophily *Anopheles gambiae s.l*. constitutes the main vector of malaria and the target of vector control programmes in Africa [1]. As a result, anopheles mosquito has developed resistance to the different classes of insecticides used in vector control stategies through two major mechanisms. The first mechanism is metabolic resistance, which is due to the increase of expression level or activity of detoxifying enzymes, belonging to three families: the cytochrome P450 monooxygenases, the glutathione S-transferases and the carboxyesterases [2]. The second and most studied mechanism is the target site resistance, such as the insensitive acetylcholinesterase resistance conferring a cross-resistance to carbamates and organophosphates by reducing the ability of these compounds to inhibit acetylcholinesterase in nerve synapses [3]. Two amino acid substitutions in the protein have been shown to play a role in resistance but the resistant allele encoding the G119S mutation, namely *ace-1^R^*, was reported several times in field *An. gambiae* populations [4], [5], [6]. In *Culex pipiens quinquefasciatus*, life history traits, reproductive capacity and physiology are strongly impacted by the *ace-1* resistance because of the interference between *ace-1^R^* allele and the general functioning of the central nervous system [7], [8], [9], [10], [11]. Little information is available so far on the impact of insecticide resistance alleles affecting other traits such as host seeking, blood feeding behaviour and pathogen transmission. A regulation of some salivary proteins was reported in *ace-1* resistant *C. p. quinquefasciatus* mosquito [12]. In this study, D7 long form protein which is known to favor the blood intake was down-regulated in resistant *Culex* mosquitoes whereas some metabolic components were up-regulated in the resistant strain [13]. For *Anopheles* mosquitoes a large fitness cost entailed by the *ace-1^R^* mutation has also been hypothesized [14]. Though reports on these fitness costs are rare, they tend to confirm this hypothesis [15]. Experimental infections of *ace-1^R^* resistant *An. gambiae* with *Plasmodium falciparum* gametocytes revealed a slight variation in parasite prevalence suggesting an impact of this mutation on the parasite development [16].

In this work we focused on the effect of *ace-1* resistance on the salivary gland protein composition in *An. gambiae*. The main function of arthropod saliva is to help blood meal intake by releasing components with anti-coagulation, vasodilatation and platelet aggregation properties [17]. In addition, this saliva at the interface with the host could strongly influence both the human immune response and the pathogen transmission. Mosquito salivary proteins play a major role in host-vector interaction and it has been suggested that salivary content could be influenced by age, or infection status of the *Anopheles* vector [18]. Other studies have reported that salivary components could intervene in pathogen transmission [19], [20]. Using a proteomic approach based on two-dimensional gel electrophoresis followed by mass spectrometry identification, we investigated whether the expression of salivary proteins would be modified in the resistant malaria vector. We performed a differential analysis of the sialome of *ace-1^R^* homozygous *An. gambiae* mosquito and its susceptible counterpart, having a similar genetic background. We therefore, describe and discuss the differential expression of the proteins identified as down- or up-regulated in the insecticide-resistant mosquito. We report for the first time differences in the composition of the insecticide-resistant anopheles sialome with differential expression of proteins belonging to a family exclusively expressed in anophelines. The known or putative functions of the regulated proteins in the invasion of salivary glands by the parasite or in blood meal intake by females are discussed in regards to how they could impact the vectorial capacity.

## Materials and Methods

### Mosquitoes

Two strains of *Anopheles gambiae* S molecular form were used in this study. The insecticide-susceptible strain Kisumu (Kis) originating from Kenya [21] is homozygous for the *ace-1^S^* allele at the *ace-1* locus, while the *ace-1^R^* Kisumu strain (AceRKis) is homozygous for the resistant allele *ace-1^R^*, bearing the G119S mutation. This strain was obtained through 19 generations of back-crossing and selection, allowing introgression into the genome of the Kis strain [5]. Female mosquitoes aged 2-3 days were fed on rabbits, then maintained on a diet of glucose until salivary gland dissection between day 8 to 10 post blood meal. The protocol for mosquitoe feeding on rabbits was approved by the animal ethics committee of Languedoc Roussillon, France (Permit number CEEA-LR-13002).

### Salivary gland extract preparation

Salivary glands were pooled (15 to 20 pairs) in 30 µl of rehydratation buffer containing 7 M urea, 2 M thiourea, 4% CHAPS (3-[(3-Cholamidopropyl)dimethylammonio]-1-propanesulfonate), 0.2% tergitol, 20 mM Dithiothreitol, 1% octylβglucoside, 0.8% IPG (Immobilized pH gradient) buffer (PlusOne reagents, GE Healthcare Bio-Sciences, Uppsala, Sweden) and stored at -80°C. After several cycles of frosting-defrosting on liquid nitrogen, samples were centrifuged at 30,000 g for 15 min at 14°C. Soluble salivary proteins in the supernatant were quantified using a Bradford Protein assay (Bio-Rad Laboratories, Marnes-la-Coquette, France) compatible with the presence of urea in the samples. The protein standard curve was run in the same buffer as the SGE samples, 2 µl of rehydratation buffer being diluted in 24 µl standard assays, to compensate for interfering components from the rehydratation buffer on the protein assay. Protein concentration was estimated at an average of 5.0 ± 0.6 µg (mean ± standard deviation) per 10 gland pairs. Total protein content of the salivary glands was slightly lower in the Kis strain compared with the AceRKis strain (4.8 µg and 5.2 µg mean values, respectively). Six samples of salivary gland extracts (SGE) containing 15 µg of proteins were prepared for each strain. Four of them were used for the analytical gel replicates while the last two samples were pooled (30 µg) for loading onto the preparative gel.

### Two-dimensional gel electrophoresis (2-DE)

SGE (15 µg) were diluted to 180 µl by adding rehydratation buffer and 2% final DeStreak reagent (GE Healthcare) and loaded onto 11 cm Immobiline DryStrip gel pH 3-11 non linear (NL) (GE Healthcare). Strips were rehydrated at 20°C overnight (16 hours) and run using the following conditions: temperature 20°C; current 50 µA per strip; 60 V for 1 hour, 500 V (gradient) for 1 hour, 1000 V (gradient) for 1 hour, 6000 V (gradient) for 2 hours, and a 6000 V step up to 30000 Vhrs. Reduction and alkylation steps were performed under standard conditions [12]. The second dimension was carried out on 10-20% SDS-PAGE gels (Bio-Rad) at 30 V for 16 min followed by one hour at 200 V. After fixation, the gels were stained with LavaPurple Protein Gel & Blot Stain Kit (Fluorotechnics, Australia) according to manufacturer's instructions. Gel scanning was performed using a Typhoon 9400 imager (GE Healthcare). Preparative 2-DE gels for spot picking were carried out in the same conditions, using 30 µg of SGE. Preparative gels were stained overnight with colloidal coomassie blue (Fermentas, Saint-Remy les Chevreuse, France) and scanned using a Perfection V750 Pro scanner (EPSON). Four analytical gels and one preparative gel were run for each strain.

### 2-DE gel analysis

Progenesis SameSpots 4.1 software (Nonlinear Dynamics, Newcastle upon Tyne, United Kingdom) was used for 2-DE gel comparative analysis. Statistical tests were performed as recommended by the software provider including a Principal Component Analysis (PCA) and an ANOVA test adjusted by a False Discovery Rate (FDR) approach for all spots in both groups (Kis and AceRKis) assigning to each spot a p-value and a q-value. The statistical power of the analysis was greater than 0.8. Spots under the ANOVA test p-value of 0.01 and above the 1.5-fold minimal difference in expression levels in either direction (up and down-expression) between the two strains were selected and manually excised from the preparative gels.

### Mass spectrometry and protein identification

Tryptic digestion of in-gel proteins was performed according to the Shevchenko modified protocol [22]. Digested proteins were dehydrated in a vacuum centrifuge, solubilised in 10 µl of formic acid (2%), desalted using Zip Tips C18 (Millipore, Bedford, MA), eluted with 10 µl of 0.1% trifluoroacetic acid in 50% acetonitrile and concentrated to 3 µl volume. 0.4 µl of analyte solutions was mixed with the same volume of alpha-cyano-4-hydroxy-trans-cinnamic acid (saturated solution was prepared in 0.1% trifluoroacetic acid in 50% acetonitrile, vortexed, sonicated 30 s and microfuged 30 s, then a 1/3 dilution of the supernatant was used as the matrix). The mixture was deposited on a 384-well MALDI target using the dry-droplet procedure [23], then air dried at room temperature.

MALDI-TOF MS analyses were performed using an UltraFlex I MALDI TOF-TOF mass spectrometer (Bruker Daltonics, Bremen, Germany) in the reflectron mode with a 26 kV accelerating voltage and a 50 ns delayed extraction. Mass spectra were acquired manually using FlexControl version 3.0 software (Bruker Daltonics) (laser power ranged from 35 to 50%, 600 shots). Spectra were analyzed using FlexAnalysis version 3.0 software (Bruker Daltonics) and calibrated internally with the autoproteolysis peptides of trypsin (m/z 842.51, 1045.56, 2211.10). Peptides were selected in the mass range of 900–3000 Da. Peptide Mass Fingerprint identification of proteins was performed by searching against the Insecta entries of Swiss-Prot and TrEMBL databases (UniProt: http://www.uniprot.org/) and by using the MASCOT version 2.3 algorithm (MatrixScience: http://www.matrixscience.com/) with trypsin enzyme specificity and one trypsin missed cleavage allowed [24]. Oxidation was set as variable methionine modification for searches. A mass tolerance of 50 ppm was allowed for identification. Matching peptides with one missed cleavage were considered as pertinent when there were two consecutive basic residues or when arginine and lysine residues were in an acidic context. MASCOT scores higher than 70 were considered significant (p<0.05) for Swiss-Prot and TrEMBL database interrogations.

Peptide samples that could not be identified by MALDI-TOF MS analysis were subjected to nanoLC ESI MS/MS analysis. Samples were dehydrated in a vacuum centrifuge, solubilized in 2 µl of 0.1% formic acid-2% acetonitrile and analyzed online by nano-flow HPLC-nanoelectrospray ionization using a LTQ Orbitrap XL mass spectrometer (LTQ Orbitrap XL, Thermo Fisher Scientific, San Jose, CA) coupled with an Ultimate 3000 HPLC (Dionex, Amsterdam, Netherlands). Desalting and pre-concentration of samples were performed on-line on a Pepmap precolumn (0.3 mm×10 mm). A gradient consisting of 0-40% A in 30 min, 80% B in 15 min (A = 0.1% formic acid, 2% acetonitrile in water; B = 0.1% formic acid in acetonitrile) at 300 nl/min was used to elute peptides from the capillary (0.075 mm×150 mm) reverse-phase column (Pepmap, Dionex). LC-MS/MS experiments comprised cycles of 5 events; an MS1 scan with orbitrap mass analysis at 60000 resolution followed by CID of the five most abundant precursors. Fragment ions generated by CID were detected at the linear trap. Normalized collision energy of 35 eV and activation time of 30 ms were used for CID. All Spectra were recorded under positive ion mode using the Xcalibur version 2.0.7 software (Thermo Fisher Scientific). Spectra were acquired with the instrument operating in the information-dependent acquisition mode throughout the HPLC gradient. The mass scanning range was m/z 400-2000 and standard mass spectrometric conditions for all experiments were: spray voltage, 2.4 kV; no sheath and auxiliary gas flow; heated capillary temperature, 200 °C; capillary voltage, 40 V and tube lens, 120 V. For all full scan measurements with the Orbitrap detector, a lock-mass ion from ambient air (m/z 445.120024) was used as an internal calibrant as described [25].

All MS/MS spectra were searched against the Insecta entries of Swiss-Prot and TrEMBL databases (release 2010_11) by using the Proteome Discoverer version 1.2 software (Thermo Fisher Scientific) and MASCOT version 2.3 algorithm with trypsin enzyme specificity and one trypsin missed cleavage. Oxidation was set as variable methionine modification for searches. A peptide mass tolerance of 5 ppm and a fragment mass tolerance of 0.5 Da were allowed for identification. Validation of mass spectrometry data was carried out using Proteome Discoverer software (p<0.01 for 2 peptides or more/protein).

## Results

### Differential analysis between SGE samples from resistant and susceptible mosquitoes

**Figure 1:**
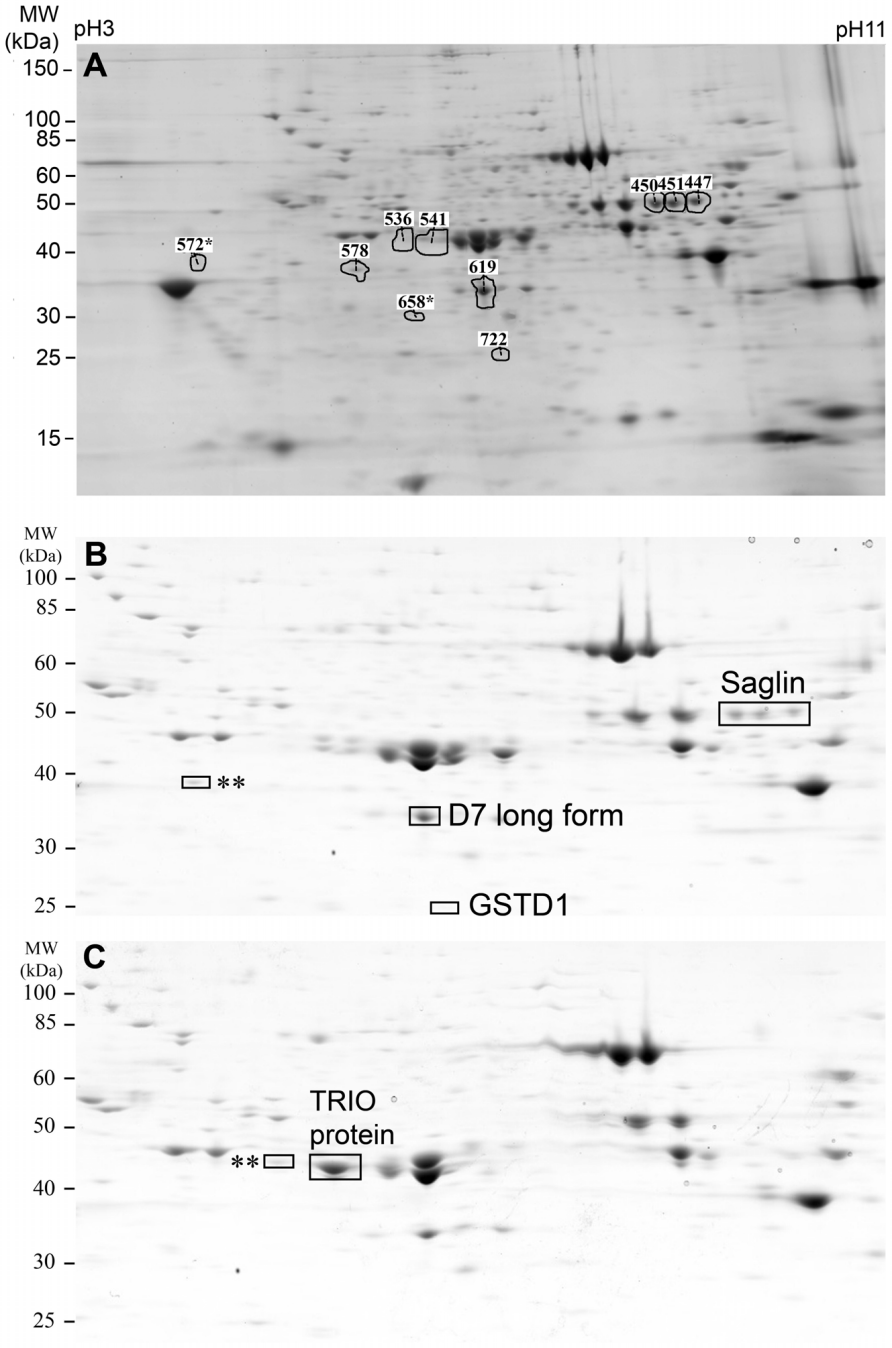
2-DE analysis of salivary gland extracts from susceptible Kis and resistant AceRKis *An. gambiae* strains. (A) Comparative analysis and statistical tests were performed using Progenesis SameSpots software. Localization of the 10 spots under the ANOVA test p-value of 0.01 and above the 1.5-fold minimal difference in expression levels between the two groups of gels are indicated on the reference image corresponding to one of the Kis SGE extract gels. *Low intensity spots 572 and 658 could not be excised from the preparative gel. (B) Identification of the differentially expressed salivary proteins excised from the susceptible Kis and (C) resistant AceRKis gels. Protein identification was performed using MALDI-TOF MS or nanoLC ESI MS/MS analysis. The three isoforms of saglin, down-regulated in the AceRKis strain, and the TRIO protein, up-regulated in the AceRKis strain, correspond to spots 447, 450, 451 and 541, respectively. **These spots were assigned to two or three accession numbers corresponding to different proteins (see Table 1).

Four independent biological repeats corresponding to four SGE samples of each *An. gambiae* strain, AceRKis and Kis, were used for the comparative analysis. Principal component analysis (PCA) separated eight gels according to expression variation into two distinct groups corresponding to the SGE samples of the two *An. gambiae* strains (PCA values of 56% and 12% for the two first components). A significant differential expression between the two strains was observed in 10 of the 708 spots according to ANOVA test (p<0.01) and a display of 1.5-fold minimal difference in expression levels. Figure 1A shows the localization of the 10 spots identified as differentially expressed by SameSpots analysis. Of these spots eight were excised from the preparative Kis and AceRKis gels, whereas the other two spots were too faint for excision (spots 572 and 658). Statistical results for the 8 spots are summarized in Table 1.

**Table 1:** Differentially expressed salivary gland proteins between the susceptible Kis and resistant AceRKis strains.

Spot#	Protein ID	p-value	q-value	Fold	Accession	MW (kDa)	pI	Cover	Score*
541	TRIO protein ^SP^	2.18×10^−5^	0.015	3.72	Q7PUJ5	43.76	6.03	35	-
					Q8WR22	43.72	6.03	33	-
447	Saglin ^SP^	1.26×10^−4^	0.023	6.38	Q2TLV8	47.10	7.66	30	91
450	Saglin ^SP^	1.59×10^−4^	0.023	4.94	Q2TLV8	47.10	7.66	33	124
451	Saglin ^SP^	2.30×10^−4^	0.031	5.83	Q2TLV8	47.10	7.66	31	131
722	GSTD1	7.17×10^−4^	0.080	4.91	Q93113	23.40	6.34	18	-
536	SG1-like 3 *^SP°^*	1.85×10^−3^	0.178	2.68	Q8WR33	30.95	5.94	39	81
	TRIO ^SP^				Q7PUJ5	43.76	6.03	29	78
578	Transaldolase	2.93×10^−3^	0.247	3.57	Q7PZ95	36.90	6.29	12	-
	Phosphatase				Q7PD38	35.50	5.54	8	-
	Translation IF				Q7PP77	36.20	5.50	8	-
619	D7 long form ^SP^	7.69×10^−3^	0.432	1.90	Q7PJ76	35.57	5.64	34	106

Protein identification was performed using MALDI-TOF MS or nanoLC ESI MS/MS (underlined spot#) analysis. Insecta entries of Swiss-Prot and TrEMBL databases were searched by using the MASCOT algorithm, or the Proteome Discoverer software for nanoLC ESI MS/MS spectra. All spots displayed a power >0.9. Spot# refers to the SameSpots analysis spot number (see Figure 1A); ^SP^ indicates the presence of a signal peptide for secretion as predicted by SignalP 4.0 (*^SP°^*: SP in the N-terminal homolog Q5TV62_ANOGA); Fold: fold difference in expression levels between the two strains; Accession: accession number in UniProtKB/Swiss-Prot or UniProtKB/TrEMBL databases (_ANOGA); pI: isoelectric point; Cover: indicates the amino acid coverage (%); *Mascot Score is provided for MALDI-TOF protein identification; for nanoLC ESI MS/MS based identification the peptide sequences and the number of peptides are provided as supporting information (Table S1). Translation IF: Translation Initiation Factor.

### Identification of the differentially expressed salivary proteins in the resistant AceRKis mosquitoes

The eight spot samples excised from the preparative gels were analysed by MALDI-TOF MS. A unique protein identification in the UniProtKB/Swiss-Prot or UniProtKB/TrEMBL database was assigned to four spots (Table 1), three of which were identified as isoforms of saglin [Q2TLV8_ANOGA)] and one as a long form D7 salivary protein [Q7PJ76_ANOGA]. A fifth spot was assigned to two proteins, the salivary gland 1-like 3 (SG1-like 3) [Q8WR33_ANOGA] and the TRIO protein [Q7PUJ5_ANOGA]. The three remaining spot samples were subjected to nanoLC ESI MS/MS analysis. This analysis allowed for the identification of the TRIO protein [Q7PUJ5_ANOGA] and an isoform of the Glutathione S-transferase 1 [Q93113_ANOGA]. On the other hand, the remaining spot sample (IDs 578) could not be assigned to a single protein. Results of protein identification using mass spectrometry are summarized in Table 1. The peptide sequences and the number of peptides detected by nanoLC ESI MS/MS and used for protein assignment are provided as supporting information (Table S1).

**Figure 2:**
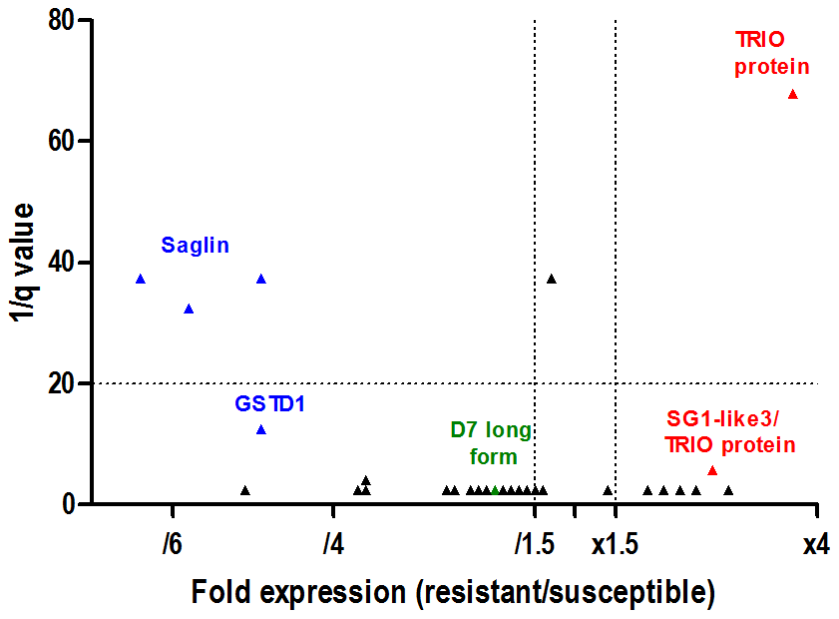
Differential expression of the salivary proteins between the susceptible Kis and resistant AceRKis SGE extracts. Differences in protein expression are represented as a function of both expression ratio (resistant/susceptible) and significance ratio (q-value). Vertical dotted lines indicate the 1.5-fold difference in expression level in either direction (x1.5 for a higher expression in the resistant strain and/1.5 for a lower expression in the resistant strain). The horizontal dotted line indicates a q-value  = 0.05 (or 1/q  = 20). Saglin and TRIO proteins display expression level differences above 3 and highly significant q-values.

Saglin, D7 long form and Glutathione S-transferase D1 (GSTD1) proteins were identified as down-regulated in the salivary glands of the resistant AceRKis strain, while the TRIO protein was up-regulated in the resistant strain (Figure 1, B and C). Figure 2 illustrates the differential expression of the identified proteins between the two strains. The graph distinguishes saglin and TRIO proteins, which displayed high expression level differences and the lowest q-values. Saglin, TRIO and D7 long form are secreted proteins as evidenced by the presence of a signal peptide which is indicative of secretion through the classical ER-golgi pathway, as predicted by SignalP 4.0 [26].

## Discussion

The approach combining comparative 2-DE gel analysis and mass spectrometry identification allowed for the identification of five proteins regulated in the resistant AceRKis salivary gland extracts, two of them showing high differences between strains. Among these proteins, saglin presented the highest differences in protein expression with an average value of 5.7 fold for the three isoforms. Saglin was first identified as the target of monoclonal antibodies that reduce salivary gland infectivity of *Plasmodium* sporozoites, suggesting that it may represent one of the molecules involved during the invasion of salivary glands by sporozoites [27]. This 100 kDa-salivary protein was described as a disulphide bond-linked homodimer of ∼50 kDa subunits, and then reported as the receptor for sporozoites in salivary gland, binding to the thrombospondin-related anonymous protein (TRAP) expressed in sporozoites and conserved in all *Plasmodium* species [28]. TRAP was shown to be essential for sporozoite gliding, cell invasion and in vivo infectivity, thus candidate target protein for malaria transmission-blocking vaccine [29], [30]. Previous sialome studies showed an over-expression of saglin in *An. gambiae* females versus males [31].

Blast analysis identified the closest homologous and orthologous sequences in *An. stephensi*, *An. cracens* and *An. gambiae* (49, 47, and 32% identity, respectively), and confirmed saglin as being a member of the salivary gland-1 (SG1) protein family, described as unique to anophelines [32], [33]. Sequence analysis of saglin did not reveal any functional domain, but an N-terminal signal peptide for ER-golgi secretion pathway and one putative N-linked glycosylation site. The three saglin isoforms identified on the 2-DE gels are thus likely unglycosylated and partially to totally glycosylated forms of the ∼50 kDa subunits of the protein. A similar hypothesis of an heterogeneous mixture of unglycosylated and partially glycosylated forms of saglin was reported from 2D Western blot analysis and treatment with peptide N-glycosidase F (PNGase F) of the recombinant protein [27]. No transmembrane domain or GPI-anchoring signal indicative of cell surface protein localization could be identified. Thus, saglin appears to be a secreted extracellular protein. Whether saglin is partly present in the lumen of salivary glands and saliva remains to be determined, since experimental evidence supports the presence of saglin on the outer surface of the distal lobes of salivary gland, facing the hemocoel [34]. Sporozoite TRAP-domain A interaction with salivary gland is mediated by binding to saglin, but it is still not clear how these proteins interact and how the surface attachment could be promoted [28]. We speculate that saglin present at the basal side of the salivary gland cells could interact with other membrane bound proteins on the basal salivary-gland surface and facilitates sporozoite interaction and salivary gland invasion. Though, more than one salivary gland protein is likely to be involved during salivary gland invasion by sporozoites, a facilitation of salivary gland invasion in the strain over-expressing saglin can be envisioned. Whether this down-regulation in the resistant strain can be linked to a lesser salivary gland invasion property by the *Plasmodium* parasite should be carefully considered. With salivary gland invasion being an essential step of the *Plasmodium* life cycle in its mosquito vector, the insecticide resistant AceRKis strain might be less susceptible to *Plasmodium* infectivity. A recent study on the vectorial competence of *ace-1* resistant *An. gambiae* for *Plasmodium falciparum* reported a low increase in prevalence of infection in the resistant mosquito regarding the oocyste stage in the midgut but not the sporozoite stage in the head and thorax [16]. The role of saglin in salivary gland invasion could thus be one factor contributing to the control of sporozoite load in this compartment. Further studies targeting both the parasite quantification in the salivary glands and the characterization of saglin partners likely to participate in salivary gland invasion by the pathogen would shed some light on the relevance of the differential expression of saglin in the susceptibility to parasite infection of *An. gambiae*.

In contrast with saglin, the TRIO protein presents a significant up-regulation in the insecticide resistant AceRKis strain. cDNA encoding this protein was identified among the salivary gland transcripts of *An. gambiae* adult females [35]. Expression data showed an over-expression of *An. gambiae* TRIO in adult female versus male, in salivary gland versus whole body extract, and in non-blood-fed versus blood-fed female [31], [36]. This female-enriched salivary gland gene product is thus likely to play a role in blood feeding, but not in the digestion of the blood meal. Rather it could be involved in the blood feeding process in relation either with anti-hemostatic/immunomodulation properties or with the different steps preceding the blood meal *i.e.* probing and blood uptake. Thus up-regulation observed in the resistant strain could testify for some differences in blood meal uptake between the sensitive and resistant mosquitoes. Sequence alignments and similarity searches performed in the present study indicate that this secreted protein is another member of the SG1 family sharing only 16% identity with saglin. Biochemical studies targeting secretion properties, localization, and function of TRIO could shed light on its role in the salivary glands of *Anopheles*, and on the significance of its up-regulation in the biology of the insecticide-resistant *An. gambiae* strain.

Surprinsingly a Glutathione S-transferase was found as down-regulated in the resistant AceRKis strain. Glutathione S-transferases (GSTs) are a diverse family of enzymes, playing a central role in detoxification of both endogenous and xenobiotic compounds [37]. *An. gambiae* GSTs have been studied for their implication in resistance to the organochlorine insecticide DDT. Expression profiling experiments identified members of the epsilon-class of GSTs but none of the delta-class GSTs as upregulated in DDT resistant strains, and functional studies confirmed the high DDT-metabolizing ability of one GST member of the epsilon-class [38], [39]. The GST identified in the present study is a member of the delta class, named GSTD1 or agGSTd1-6 [39], [40]. Structural studies revealed differences in the substrate-binding site conformation that explained the very low DDT-detoxifying activity of this GST [41], [42]. Thus, the down-regulation of the protein in the resistant strain might not be linked to a decreased insecticide detoxifying activity, but the relevance of this difference in expression observed between the two strains is unclear. Besides their detoxifying function GSTs are involved in a wide range of biological processes including protection against oxidative stress [43]. Their contribution to the removal of toxic oxygen free radicals and maintainance of the redox status of the cell represents a function that deserves further attention to better understand the role of this enzyme family, particularly in relation to parasite infection and vector competence.

Although the down-regulation of the secreted D7 long form protein observed in the resistant strain is low, this protein deserves some comment. Indeed, its ortholog in *Culex pipiens quinquefasciatus,* named long form D7 cluster12 salivary protein, was previously recorded as differentially expressed between the *ace1-^R^* insecticide-resistant and the susceptible strains [12]. D7 protein family is widely conserved in blood sucking diptera, and its expression is restricted to female salivary glands suggesting a potential role in the blood meal process [44]. This family is ancestrally related to Odorant Binding Protein family, but is a separate branch as supported by the structural analysis of *Anopheles* D7 proteins members [13], [45]. The *Anopheles* D7 family is composed by three long form proteins (27-30 kDa) and five short form proteins, named D7r1-D7r5. The short forms (D7r1-r4) have been demonstrated binding biogenic amines, like serotonin, norepinephrin and histamine, highlighting functions in the inhibition of itching, burning and vasoconstriction induced by the bite [13]. D7r1 possess also anticlotting activity, as its ortholog in *An. stephensi*, hamadarin. Function of the *Anopheles* species D7 long form recently became elucidated. A structure/function study on the long form D7 L1 from *An. stephensi* has shown that this protein binds cystenyl-leukotrienes and analogues of tromboxane A2. These molecules are released during early inflammatory response following injury, contributing to pain, increase of vascular permeability and platelet activation in the host [46]. The high similarity observed between the D7L1 and the *An. gambiae* ortholog (60%), especially for amino acid residues involved in ligand binding pocket (80% identity), strongly suggests that the *An. gambiae* D7 long form could have similar functions in blood feeding. The down regulation of this D7 long form protein may thus alter the efficacy of the blood feeding process, with less inhibition of platelet activation and anticlotting activity of the host. The blood feeding might be interrupted hence leading the mosquito to bite several hosts/times to achieve a complete blood meal. Biting behavioural studies comparing resistant and susceptible strains of *An. gambiae* as well as RNAi studies would help to determine the implication of this D7 long form protein in the blood feeding success.

The resistant AceRKis strain was obtained by introgression of the *ace-1* G119S allele (*ace-1^R^* allele) from a resistant population into the Kisumu genome. After 19 generations of introgression, the two *An. gambiae* strains should share the same genetic background for most of their genome, mainly differing in their genotype at the *ace-1* locus: the Kis strain is homozygous for the *ace-1^S^* allele and the AceRKis strain is homozygous for the *ace-1^R^* allele. Comparative studies of the two strains should thus reveal changes in characters or phenotypes that could be essentially attributed to the G119S mutation. Nevertheless, the genetic differences between the two strains might not be totally negligible: the presence of inversions, founder effects, and the genes closely linked to *ace-1* could have prevented part of the expected recombination. Regarding chromosomal inversions, differences in the parental strains could hamper recombination and limit introgession [47]. Indeed, loci inside a polymorphic inversion and in linkage disequilibrium with the resistant allele may be selected with this allele. The origin of the Kisumu strain is East Africa, where *An. gambiae* is polymorphic for the chromosomal inversions 2Rb and 2La [48]. The insecticide resistant parental strain was colonized from wild mosquitoes from Bobo-Dioulasso (Burkina Faso) where the S form of *An. gambiae* has been shown to also possess the 2Rb and 2La inversions at high frequency [49]. Thus, as the two parental populations share the same inversions, they should not prevent recombination between the two strains, except if a founder effect randomly fixed different inversions before or during the introgression process. Moreover, as the *ace-1* gene is located in the telomeric region of the chromosome 2R (region 7C), thus far from the closest inversion (2Rb, between sections 11C and 13A), these inversions are not expected to affect recombination near this gene, so that the inversions present in Kis should have been introgressed in AceRKis. If Kis was polymorphic at the beginning of the backcrosses, a unique variant of some genes could have been fixed in AceRKis, due to founder effect, while remaining polymorphic in Kis, thus inducing differences between the two strains. However, as each generation required a new backcross on Kis, this effect would be relatively limited, as most of the Kis polymorphism would have passed in AceRKis. We cannot however rule out the possibility that other genes, such as regulatory regions and loci physically linked to *ace-1^R^* could have hitch-hiked with it during the introgression process. Indeed, at the risk level of 0.05, a genetic distance of 15 cM around *ace1^R^* would not have been replaced during the successive backcrosses [5], [50]. According to the average relationship between genetic distance and physical distance on the chromosome X [5], [50], that would correspond to approximately 11 Mb of DNA that could differ between the two strains, depending on the initial polymorphism. In conclusion, we cannot exclude that part of the differences in salivary protein expression observed in the present work between Kis and AceRKis could be due to cis-regulatory elements or other loci from the genome in which the *ace-1^R^* mutation originally occured, rather than to the mutation itself. The analysis of the non-synonymous polymorphism between the two strains, particularly in the flanking regions of the *ace-1* locus, could help in quantifying the real impact of the introgression process. Finally, additional crossing experiments between the two strains followed by the analysis of the co-segregation of salivary protein expression and the resistance gene are required to formally establish the causal relationship between the regulation of the salivary protein genes and the resistance allele.

## Conclusion

The present work is the first attempt to determine whether insecticide-resistance in *An. gambiae* mosquitoes could induce a modulation of protein expression in the salivary glands and have some consequences on blood feeding, trophic behaviour and on *Plasmodium* transmission. We describe for the first time modifications in the sialome composition of insecticide-resistant *An. gambiae*. The known or putative functions inferred from sequence similarity analyses revealed that the differentially regulated proteins identified in this study are involved in pathogen invasion, blood feeding process, or protection against oxidation. These proteins deserve functional studies to understand the relevance of the differences in expression recorded in the insensitive acetylcholinesterase resistant mosquitoes. This data confirms the profound alterations linked to the resistance in the anopheline mosquito biology but additional genetic studies on the polymorphism between the two strains are needed to confirm whether *ace-1^R^* allele is responsible for the variation in protein expression and to determine whether the expression levels influence phenotypes relevant for transmission. Experiments on field mosquitoes bearing the *ace-1^R^* allele or the *ace-1* gene duplication [6] could also help to specify the link between *ace-1* alleles and phenotypes. Further experiments on the behaviour of these mosquitoes during blood feeding will contribute to the understanding of the impact of resistance on blood meal intake and on the transmission of the pathogen. This work opens the way for studying the profound biological alterations in the mosquito linked to the different insecticide resistance mechanisms, alone or in combination.

## Supporting Information

Table S1 **Details of the nanoLC ESI MS/MS based identification of the differentially expressed salivary gland proteins in insecticide-resistant AcerKis strain.** Protein identification was performed using nanoLC ESI MS/MS analysis. Insecta entries of Swiss-Prot and TrEMBL databases (release 2010_11) were searched by using the Proteome Discoverer software. Spot # refers to the SameSpots Analysis spot number (see Figure 1A); Accession number: accession number or entry name in UniProtKB/Swiss-Prot or UniProtKB/TrEMBL; In the peptide sequence column, m means methionine oxydation.
(XLSX)
