# Correction: Salivary Gland Proteome Analysis Reveals Modulation of Anopheline Unique Proteins in Insensitive Acetylcholinesterase Resistant *Anopheles gambiae* Mosquitoes

**DOI:** 10.1371/journal.pone.0113440

**Published:** 2014-11-14

**Authors:** The PLOS ONE Staff

The affiliation for the 11^th^ author is incorrect. Françoise Mathieu-Daudé is not affiliated with #1 but with #4 Maladies Infectieuses et Vecteurs, Ecologie, Génétique, Evolution et Contrôle (MIVEGEC), UMR IRD 224-CNRS 5290-UM1-UM2, Institut de Recherche pour le Développement (IRD), Nouméa, Nouvelle-Calédonie.
